# Development of a program to prevent sexual violence among teens in Japan: education using DVD video teaching materials and web-based learning

**DOI:** 10.1186/s12199-021-00964-y

**Published:** 2021-03-26

**Authors:** Miyuki Nagamatsu, Narumi Ooshige, Nozomi Sonoda, Mika Niina, Ken-ichi Hara

**Affiliations:** 1 Department of Maternal and Child Nursing, Japanese Red Cross Kyushu International College of Nursing, 1-1 Asty, Munakata City, Fukuoka 811-4157 Japan; 2 Graduate School of Medical Science, Saga University, 5-1-1 Nabeshima, Saga City, Saga 849-8501 Japan; 3 Domestic Violence Countermeasures Prevention Center Kyushu, 2-5-15 Tojin, Saga City, Saga 840-0813 Japan

**Keywords:** Adolescents, Learning, Prevention, Sexual violence

## Abstract

**Background:**

This study aimed to develop an education system using DVD video-based teaching materials or web-based learning to reduce sexual violence among teens in Japan.

**Methods:**

During the first stage, June 2018 to March 2019, an education program using DVD video teaching materials was carried out at three high schools and four universities with research consent from the director of the facility. From 1337 high school students and first- and second-year university students, subjects in their teen years were targeted for analysis. A survey was conducted at baseline and after the DVD video teaching. During the second stage, November 2019 to March 2020, web-based learning using improved video teaching materials was developed and carried out. From the adolescents who participated in the web-based learning, subjects in their teen years were targeted for analysis. A survey was conducted at baseline and after the web-based learning.

**Results:**

In the first stage, 876 students consented to and participated in the education using DVD video teaching materials and baseline and after surveys (collection rate 65.5%). Among these, the number of respondents in their teens both baseline and after education was 705 persons (valid response rate 80.4%). In the second stage, the number of respondents in their teens both baseline and after education was 250 respondents in their teens who received web-based learning using the improved video teaching materials (valid response rate 87.1%). The improvement effect of the two programs was observed in attitudes that lead to physical violence, attitudes that lead to mental violence, attitudes that promote healthy conflict resolution, and dangerous attitudes that lead to sexual violence from persons in the community or through the Internet. The web-based learning program achieved an improvement of preventive attitudes toward sexual violence.

**Conclusions:**

The education program using DVD video teaching materials or web-based learning may help prevent sexual violence among teens in Japan.

## Introduction

### International definition of violence

The World Health Organization (WHO) has recognized sexual violence as an important international health and human rights issue and recommends strengthening preventive measures [1]. Sexual violence is defined as sexual activity that is non-consensual, unequal, or coerced and includes sexual abuse, such as sexual assault and indecent assault, and sexual intimate partner violence [1]. In order to prevent a person from becoming either a perpetrator or victim of violence, education strategies on violence prevention are of utmost importance. Research, implementation, and assessment of education programs are required.

### Actual conditions of violence in Japan

In Japan, the Domestic Violence (DV) Prevention Act was revised to allow protection orders to be issued also for cohabitating dating partners. Additionally, the Anti-Stalking Act was revised to regulate repeated sending of emails [2]. In 2015, after the implementation of the Anti-Stalking Act and the Act on the Prevention of Spousal Violence, the number of stalking cases and spousal violence cases in Japan increased dramatically [3]. In 2017, 21.4% of women and 11.5% of men were reported to have experienced violence from a partner [4].

In recent years, the percentage of adolescents with smartphones with daily access to the Internet has increased: approximately 70% of junior high school students and 97.5% of high school students [5]. Previous studies in Japan demonstrate that the factors which lead to the risk of harm by sexual violence involving adolescents are related to behaviors such as sending messages and pictures to persons they first contacted online, attitudes that accept sexual activity, and low awareness of DV [6]. Furthermore, according to the 2015 National Police Agency report, the number of recognized cases of indecent assault for both men and women was 4129 cases, the highest number of cases since the indecent assault and public indecency began to be classified separately in 1966 [7]. Since 2015, cases have continued to increase, with the number of injury cases involving indecent assault increasing to 4320 cases [8]. In 2017, the classification of rape offenses, which had been limited to women, was revised to coerced sexual assault and, based on the Gender Equality Act, also included male victims. The reported number of coerced sexual assaults was 1027 cases [8]. Of these, 57% of the perpetrators of coerced sexual intercourse were acquainted with the victim [8]. However, the actual number of offenses is presumed to be higher due to the barriers to making a complaint.

### Theoretical background and research for the international prevention of sexual violence

Cohen and Felson proposed the routine activity approach, in which crime stems from three factors: a motivated offender, a suitable target, and the absence of a capable guardian [9]. Gottfredson and Hirschi also proposed the self-control theory suggesting that crime is more likely to occur as a result of lower self-control and that women with lower self-control are more likely to suffer sexual violence [10]. Additionally, studies in the USA showed that sexual violence toward female college students was significantly associated with low self-control and daily routine activities [11]. The psychosocial abilities required to constructively and effectively cope with the various demands and problems arising in daily life are defined as life skills. Within these, social skills focusing on basic communication is the foundation on which better relationships with others are built; hence, communication-based social skills training is necessary for healthy relationships [12]. In the USA, Ball et al. used a violence prevention program carried out by a support group for junior high and high school students who were at high risk of becoming perpetrators or victims of violence [13]. They reported that a reduction in problems in male-female relationships and friendships and an improvement in skills to build healthy relationships may be achieved with social skills training [13].

### Violence prevention education in Japan

While group education to increase awareness of DV and promote respectful relationships has been carried out among junior high school students [14], there are few empirical studies on violence prevention education in Japan. The principal researcher of one regional study recommended strengthening ICT-based learning as an education method for junior high school students regarding the prevention of and response to sexual violence from close associates, persons in the local community, or online [15]. However, efforts to expand the target population and the region to prevent adolescents from becoming a perpetrator or victim of violence in Japan are needed.

The objective of this study was to develop educational material for teens in Japan, using DVD video teaching materials and web-based learning, to prevent sexual violence, in particular, from intimate partners, persons in the community, or through the Internet. For the first stage, implementation of the program and assessment was carried out at junior high schools, high schools, and universities in some Japanese regions using DVD video teaching materials. Following the after surveys, the video teaching materials were refined by referring to comments and opinions from research collaborators. The objective of the second stage was to implement and assess education using a web-based learning program, which allowed users to improve their skills at their own pace regardless of location or time, through the use of improved and revised video teaching materials and personal computers or mobile terminals.

## Materials and methods

### Educational concept

To enhance the educational effects, this study used instructional design to build learning materials and processes through the introduction of the following: attention—“That seems interesting.”; relevance—“It concerns me.”; confidence—“I can do it.”; and satisfaction—“I’m glad I did it.” The theoretical concepts of the ARCS (Attention, Relevance, Confidence, and Satisfaction) model [16] and the 9 instructional events of Gagne [17] were incorporated to encourage learners to gain knowledge, skills, and attitudes. Additionally, the web-based learning video material based on instructional design, and adopting the ARCS model and Gagne’s 9 instructional events, used examples that can be specifically imagined to prevent becoming adolescents from becoming perpetrators or victims of violence from intimate partners, persons in the community, or through the Internet. Additionally, a participatory learning process in which learners can independently learn was constructed by social skills training.

In the first stage, the theme of the DVD video teaching material was determined to be “Hidden risks and enjoyment in relationships between you and me,” with a two-step learning structure. Viewing time was set at approximately 17 min for step 1 and approximately 21 min for step 2, for a total of around 38 min. Around 5 min was allotted for questions and answers at baseline and after learning.

For the second stage, the web-based learning theme using improved DVD video teaching materials was set as “Hidden risks between you and me and a give-and-take enjoyable relationship.” Viewing time was set at approximately 15 min for step 1 and approximately 17 min for step 2, for a total of around 32 min. Approximately 5 min were allotted for questions and answers at baseline and after learning (Table 1).

**Table 1:** Learning objectives and content by DVD video teaching materials and a web-based learning program

Learning objectives	Content	Time
Evaluate attitudes before learning	Answer pre-learning questions	5 minutes
Step 1 Learn about the hidden risks in relationships with someone close, as well as countermeasures and how to handle them.	Learn about the diversity of sex Learn about sexually transmitted diseases Learn about dating DV Think about equal relationships Summary of learning	DVD video: 17 minutes Web-based learning: 15 minutes
Step 2 Learn about the risks in relationships, either face-to-face or through the internet, and their prevention	Learn about sexual harm Consider the risks when sharing information with strangers through the internet Learn about preventing unwanted pregnancies Summary of learning	DVD video: 17 minutes Web-based learning: 17 minutes
Evaluate attitudes after learning	Answer post-learning questions	5 minutes

For both education using DVD video teaching materials and a web-based learning program, sexual violence prevention education was presented in 2 steps.

Step 1 was “Learn about hidden risks in relationships with someone close as well as how to develop countermeasures and how to handle them.” Topics covered were as follows: (1) Learning about diversity in sex—sex is one of our inherent characteristics. Some people may have a sexual orientation toward the same sex, and some may feel troubled due to a sense of discomfort with the mismatch between their physical appearance and sexual mindset. Students learn about diversity in sex and think about individual dignity and fair relationship with others. (2) Learning about sexually transmitted diseases—learn about the changes in the number of patients with sexually transmitted diseases in Japan, the routes of infection, and symptoms. Preventive methods are also presented. (3) Learning about dating violence—explore the status of male and female victims and understand the hidden risks in a person close by, by viewing videos of dating violence. (4) To think about a fair relationship—by watching a video in which both female and male avoid becoming a victim, students think about countermeasures to avoid becoming either an assailant or a victim of dating violence and become aware of the actions that should be taken in threatening situations. (5) Summarizing what students learned.

Step 2 was “Learn about the risks in relationships, either face-to-face or through the Internet, and their prevention”: (1) To learn about sexual crimes in the local community—the characteristics of assailants of forced intercourse and indecent assault and the number of crimes involving male and female victims. Students are also expected to understand the local community’s current status through case studies of female and male victims. (2) Students consider the risk when information is transmitted to an unknown person via the Internet. The hidden risk of the Internet is explored through video case studies and countermeasures. (3) To learn how to prevent unwanted pregnancy—students learn about pregnancy and birth control methods to avoid pregnancy. Measures that can be taken in case of sexual violence are also discussed. (4) To summarize what they learned.

### Research period and subjects

In the first stage, for 3months, between June 2018 and March 2019, education using DVD video teaching materials was carried out at three high schools and four universities with research consent from the director of the facility, such as the school principal or president in region A. Among 1337 high school students and first- and second-year university students, subjects in their teen years were targeted for analysis.

In the second stage, from November 2019 to January 2020, a web-based learning program was developed using improved video teaching materials, and an education program was conducted using these materials. From the 287 persons who participated in the web-based learning program, subjects in their teen years were targeted for analysis.

The education using DVD video teaching materials comprised almost the same content but had an in-class format. In contrast, in the web-based learning program, students could perform iterative learning anywhere throughout the day using either the mobile web or an Internet home page. Subjects for the analysis of this study were restricted to teenage students who were within the scope of this education program. Accordingly, participants aged 20 years or older were excluded from the analysis. To calculate the sample size, G*Power version 3.1.9.7 was used. A total of 428 participants (214 participants per group) were required to achieve an effect of .35, power of .95, and a significance level of .05 for the *t* test calculation.

### Survey procedures

In the first stage, schools were randomly selected from among junior high schools and universities in region A. A principal’s request form explaining the learning program using DVD video teaching materials, a teacher request form, consent forms, implementation guidelines and instructions, and user manuals and questionnaires for students were sent, and participating schools were recruited. Participation in the learning program was at the discretion of the director of the institution and that the institution could withdraw from the study at any time. At junior high schools and high schools, which had agreed to participate in the learning program and for which the principal had provided written consent, the program and the baseline and after survey were carried out with the cooperation of the teacher in charge of the class. At universities which agreed to participate in the learning program, the program and the baseline and after survey were carried out with the cooperation of faculty members, such as the Student Support Committee, Health Management/Lifestyle Guidance, and persons in charge of the academic year, curriculum, etc. according to the conditions at the university for which the President or Dean had provided written consent.

In the second stage, the contents of the web-based learning for preventing adolescents from becoming perpetrators or victims of violence targeting persons in their teen years were introduced, and participants were recruited through the websites of organizations and academic associations related to the prevention of violence, and the university website. The web-based learning could be viewed individually or in groups. The educational effect was assessed with a questionnaire at baseline and after the web-based learning program.

### Survey methods and ethical considerations

#### In the first stage: the education by DVD video teaching materials

The following information was clearly written and requested in the student manual to explain the objective of this study and obtain participants’ understanding: This questionnaire aims to learn and understand thoughts and attitudes to “Hidden risks and enjoyment in relationships between you and me” and is meant to help you improve your learning. Please answer the questionnaire once before the educational program (front) and once after the educational program (back). The results from before and after the educational program will be compared to determine the program’s effect.

The following contents regarding the question and answer methods were clearly explained in the teacher and student manuals: You are free to choose whether or not to answer any question. If you do not want to answer a question or do not know the answer, you do not have to answer. If you decide to answer a question, please circle your answer. There is no penalty for refusing to answer any question. If you agree to submit the completed questionnaire, place it in the collection bag.

The following information regarding personal privacy was explained and distributed in the student manual: Personal secrets are completely confidential. You do not need to write your name, and individuals will not be identified at any point. Your family and teacher will not see what you have written. The answers provided will not affect your school performance. The results are summarized as numerical values.

#### In the second stage: the education by a web-based learning program

The following contents were clearly described on the website: As the use of the Internet expands, violence between friends and dating partners has increased. Sexual violence is taking place through both local life and the Internet. Therefore, let us learn about the hidden dangers in human relationships in our society in two steps using the web-based learning program, “Hidden risks and enjoyment in relationships between you and me,” with a video drama featuring university students, along with explanatory information. How to answer the questions: Please take about 5 min to answer the questions before and after watching the web-based learning program. Please access the questionnaire page in advance and answer the questionnaire using a computer or mobile terminal before watching the learning material (access by Q.R. code). When watching as an individual, you can use a personal computer or mobile terminal. After viewing, reaccess the questionnaire page and answer the questionnaire.

The following contents are clearly described on the website: To match before and after questionnaires, please enter a 4-digit number such as “the last 4 digits of your mobile phone.” You are free to choose whether to answer a question or not. There is no penalty for refusing to answer any question. If you do not understand a question or do not want to answer it, you do not have to answer it. Please enter the answers that you think you can. If you want to submit the entered answer, please click the submit button. If you do not want to submit the information you entered, you do not have to click the submit button.

The following contents were clearly described on the website: You do not need to enter any personal information such as your name. The study will not identify individuals. Personal information such as your email address is not collected when the entered contents are sent. The information entered will not be seen by your family or teachers. The information entered will not affect your school performance. The results are summarized in numerical values.

When a group of many students watched the web-based learning course, the survey implementation was carried out at baseline and after the learning program using the same method used in the first stage to avoid congestion when multiple subjects accessed the Internet.

##### Survey contents

1. At the start of either education program, DVD video teaching materials or web-based learning program, age was requested as an attribute using the following question: “Please circle your age.” Subjects were requested to select from three choices, 10s, 20s, or 30 years or older.

Before and after the education programs, questions on the following three attitudes (items (2) to (4)) were asked.

(2) Attitudes that lead to violence and healthy conflict resolution

The scale developed by Ball was used to evaluate preventive education [13].

“When spending time with your boyfriend, girlfriend, or close friend, do you think about taking the following actions toward the other person? Circle one from 0 (no) to 3 (yes).” was asked.

A. Attitude that leads to the perpetration of violence

(a) Attitude that leads to the perpetration of physical violence: “hit, throw things.” (b) Attitude that leads to the perpetration of emotional violence: “break the other person’s things, yell at or belittle the other person, tease the other person in front of others, blame the other person, threaten by saying you will date someone else, change tone of speech or express hostility, say something to offend the other person.”

B. Healthy conflict resolution

(a) Empathy: “try to understand the other person’s feelings from his/her point of view, ask what the other person is thinking, listening while considering what the other person wants to say.” (b) Assertiveness: “explain your feelings to the other person, tell the person that he/she is important to you, can say no when needed.” (c) Discussion with other individuals: “calmly discuss problem-solving with the other person, suggest a solution that will make both you and the other person happy.” (d) Attack avoidance: “sometimes leave the room so you and the other person can calm down, sometimes you do not talk until you and the other person calm down.”

(3) Dangerous attitudes toward sexual violence

The following original scale (5 items) created in the 2014-28 Grant-in-Aid for Scientific Research (C) “ICT-based adolescent education for coping with and preventing sexual violence in Japan targeting junior high school students [18]” was used: “send messages to a stranger on the Internet, send selfies to a stranger on the Internet, meet a stranger on your own whom you had contacted on the Internet, meet a stranger on your own in the area where you live, meet a stranger on your own whom you only know by face in the area where you live.”

(4) Preventive attitudes toward sexual violence

When holding a safety class in 2004 on attitudes to prevent violence in the region, the youth development section of the Tokyo Metropolitan Police Department and the Education Planning Department of the Tokyo Metropolitan Government created a safety slogan with an impact that would stick in children’s minds [3]. A scale (5 items) was also developed based on this safety slogan and used in this DVD video teaching material: “do not follow strangers; do not get in a stranger’s car; if you feel like you are in danger, scream or make a loud noise; if you feel like you are in danger, quickly run away; if you feel like you are in danger, tell an adult.”

(5) Free comments and opinions about the education

After the education, students were asked, “If you have any comments or opinions about the DVD, please feel free to write them down.”

##### Statistical analysis

The average scores for attitudes that lead to violence and for each category and the average scores for attitudes that promote healthy conflict resolution and for each category were compared and analyzed at baseline and after the education program. Dangerous attitudes toward sexual violence and preventive attitudes toward sexual violence were also compared at baseline and after the learning program and analyzed by analysis of variance (ANOVA). Two-way ANOVA was employed to assess whether there was a significant interaction between measures of the effect of intervention between the baseline and after surveys stratified by education using DVD video teaching materials and the web-based learning program. The Statistical Package for the Social Sciences (SPSS 25.0) was used for these analyses, and the level of significance was set at *p*<0.05.

## Results

### Survey results baseline and after the education program using the DVD video teaching materials (Table 2)

The number of persons who gave consent and participated in the survey baseline and after the education program using DVD teaching materials was 876 (collection rate 65.5%). Among these, 705 were in their teens both baseline and after education (valid response rate 87.1%).

**Table 2:** Two-way ANOVA for the differences of the variables according to the interaction between baseline/after or education using DVD video teaching materials/the web-based learning program and each mean value

	Education using DVD video teaching materials (*n*=705)						Web bases learning (*n* = 250)						Education using DVD –Web bases learning ×	
	Baseline		After		Baseline-After ^a)^		Baseline		After		Baseline-After ^a)^		Baseline-After ^b)^	
	*M*	*SD*	*M*	*SD*	*F*	*p*	*M*	*SD*	*M*	*SD*	*F*	*p*	*F*	*p*
**Attitudes that lead to violence**	0.46	0.43	0.25	0.31	107.70	<0.001	0.36	0.41	0.15	0.24	46.24	<0.001	0.03	0.862
Attitude that lead to physical violence	0.41	0.63	0.15	0.38	87.80	<0.001	0.25	0.52	0.04	0.19	35.55	<0.001	1.06	0.303
Attitude that lead to mental violenc	0.48	0.44	0.27	0.33	88.64	<0.001	0.39	0.42	0.18	0.27	41.50	<0.001	0.02	0.883
**Attitude toward healthy conflict resolution**	2.02	0.53	2.36	0.62	111.91	<0.001	2.08	0.52	2.52	0.53	82.72	<0.001	2.75	0.097
Empathy	2.22	0.69	2.51	0.74	55.44	<0.001	2.35	0.63	2.71	0.54	45.90	<0.001	1.01	0.313
Assertiveness	2.07	0.69	2.40	0.73	68.43	<0.001	2.15	0.70	2.58	0.60	54.34	<0.001	2.40	0.121
Discussion with other individuals	2.12	0.77	2.48	0.78	75.56	<0.001	2.27	0.73	2.67	0.64	40.72	<0.001	0.18	0.672
Attack avoidance	1.52	0.88	1.69	1.05	42.99	<0.001	1.34	0.69	2.01	1.07	55.78	<0.001	10.03	0.002
**Dangerous attitudes that lead to sexual violence**	0.43	0.54	0.23	0.45	55.89	<0.001	0.26	0.50	0.17	0.36	24.19	<0.001	0.02	0.880
**Preventive attitudes toward sexual violence**	2.62	0.58	2.68	0.64	3.46	0.063	2.61	0.60	2.80	0.45	15.94	0.001	4.26	0.039

^a^The measures were compared between baseline and after the survey on each of the two programs by ANOVA

^b^The interaction between the baseline/after and two intervention surveys was analyzed by two-way ANOVA

According to the corresponding *t* test, the average score of attitudes that lead to violence decreased after the education program compared to the baseline of the program (*p* <0.001). Among these, the average scores of both attitudes that lead to physical violence and attitudes that lead to mental violence decreased (*p* <0.001). The average scores for attitudes toward healthy conflict resolution increased (*p* <0.001). Among these, average scores for empathy, assertiveness, discussion with individuals, and attack avoidance increased, as well as the results such as a lower average score of dangerous attitudes toward sexual violence from the community or person through the Internet (*p* <0.001).

### Survey results baseline and after the web-based learning program (Table 2)

The web-based learning program, using the improved video teaching materials based on the opinions and comments of study participants and who responded both baseline and after the education in the second stage, was taken by 250 adolescents (valid response rate 87.1%). They were in their teen years.

The average score of attitudes that lead to violence significantly decreased after the web-based learning program compared to baseline (*p* <0.001). Among these, the average scores of both attitudes to cause physical violence and attitude to cause mental violence decreased (*p* <0.001). Additionally, the average score for attitudes toward healthy conflict resolution increased significantly (*p* <0.001). Among these, empathy, assertiveness, discussion with other individuals, attack avoidance, and the average score of all attitudes increased. Overall, an improvement effect with the web-based learning program was observed, including a lower average score of dangerous attitudes toward sexual violence from persons in the community or through the Internet (*p* <0.001) and an increase in the average score for preventive attitudes toward sexual violence (*p* = 0.001). On the whole, the feedback provided by the students after the learning program showed a positive impression of the teaching program: negative impressions and opinions were not observed.

### Education using DVD video teaching materials and the web-based learning program (Table 2)

A significant main effect between baseline /after and education using DVD video teaching materials/web-based learning program was noted with respect to the attack avoidance (*p* = 0.002). The web-based learning program achieved an improvement of attack avoidance on attitude toward healthy conflict resolution and preventive attitudes toward sexual violence (*p* = 0.039). The mean scores of attitudes to cause violence, empathy, assertiveness, discussion with other individuals, and dangerous attitudes toward sexual violence did not show significant differences between the effect of web-based and DVD learning.

## Discussion

This study aimed to improve the attitudes that lead to mental and physical violence by providing an education program using DVD video teaching materials and web-based learning to prevent sexual violence. The education programs showed an improvement effect and resulted in an improved attitude toward promoting healthy conflict resolution. The educational programs also improved dangerous attitudes toward sexual violence from persons in the community or through the Internet.

### An improvement in attitudes that lead to mental and physical violence after taking DVD or web-based education program

Adolescents showed improvement in attitudes that lead to mental and physical violence after taking the education program using DVD video teaching materials to prevent violence. The same improvement effect was shown with the web-based violence prevention learning program. These results are in line with those of an American violence prevention program using a support group that showed that junior high and high school students could reduce violence in male-female relationships and friendships following an education program [13]. In comparison, in a study implementing violence prevention education via the Internet for junior high school boys in Japan, attitudes that lead to physical violence improved following the education program, but attitudes that lead to mental violence did not [15]. In the latter study, data on gender was not collected, and the issue of teenage gender dysphoria was not considered. However, in the overall results of adolescent study participants, an improvement effect in attitudes that lead to physical violence and attitudes that lead to mental violence was observed. A likely factor that increased the educational effect of the programs was the greater interest shown by adolescents after presenting data on how an adolescent can become a perpetrator or victim of sexual violence from persons in the community or through the Internet and the realization that this issue closely affects them.

In 2018, 39.4% of boys and 43.1% of girls were reported to have sent messages to someone they first contacted on a mobile phone or through the Internet, and 13.0% of boys and 19.6% of girls sent photos to someone they first contacted on a mobile phone or through the Internet [19]. In recent years, the use of smartphones has increased considerably among adolescents. An Internet usage environment survey of adolescents in 2016 found that the Internet usage rate of adolescents between 10 and 17 years was 80.3% for junior high school students (smartphone ownership rate 42.7%) and 97.7% for high school students (smartphone ownership rate 92.3%) [5]. By 2018, these percentages were 95.1% (smartphone ownership rate 70.6%) for junior high school students and 99.0% (smartphone ownership rate 97.5%) for high school students [5]. Utilizing the Internet to provide education programs led to an improvement in attitudes that lead to mental and physical violence, and strengthening these education programs to reduce violence should be considered a priority.

### Attitude toward healthy conflict resolution that leads to mental and physical violence after taking DVD or web-based education program

Adolescents strengthened their attitudes toward healthy conflict resolution after receiving an education program using DVD video teaching materials to prevent violence. The same effect was shown with the web-based learning program to prevent violence. Ball et al. reported that a violence prevention program carried out in the USA among high-risk high school students using a support group reduced problems in male-female relationships and friendships following the intervention, and increased skills for healthy conflict resolution [13]. In terms of attitudes toward healthy conflict resolution, a recent study reported that discussion with other individuals could be strengthened after online education on violence prevention in junior high school girls in Japan, but no effect was observed for other items [15]. In the present study, gender was not differentiated; however, overall, an improvement effect was observed in all items regarding attitudes toward healthy conflict resolution, including empathy, self-expression, discussion with others, and attack avoidance. A factor that likely impacted the improved educational effect was the DVD-based and web-based programs’ use of drama-style videos (including empathy, assertiveness, discussion with others, and attack avoidance) as life skills training. This mode of learning makes it easier for adolescents to imagine an actual situation with a visual demonstration of how bad relationships lead to violence and how good relationships lead to conflict resolution. A 2000–2010 review of US and Canadian dating DVs reported 53 risk factors and 6 protective factors. Empathy, age, IQ, academic performance, maternal and child relationships, and school relationships were indicated as protective factors [20]. Consequently, enhancement of empathy is thought to lead to the prevention of DV. A program that added education for the prevention of sexual violence in addition to DV was shown to be effective in improving discussion between close friends and men and women promoting an attitude of mutual respect and avoidance of attacks. It is presumed that this prevents them from becoming a perpetrator or victim of sexual violence. Results of this study in Japan could clarify that recognition of DV is related to the awareness of male-female relationships with mutual respect [21]. It is thought that adolescents interested in male-female relationships need to understand what actions correspond to violence and learn about relationships based on mutual respect as well as how to avoid attacks from close friends and intimate partners. Therefore, increased opportunities to learn about attitudes that promote healthy conflict resolution may reduce the chances of adolescents becoming involved in violence.

In particular, the web-based learning program achieved an improvement of attack avoidance on attitude toward healthy conflict resolution. In recent years, a study assessing DV prevention education in college students using web-based learning observed an increase in their understanding of dating DV [22]. However, this study did not include teenage junior high school students or high school students who are commonly victims of dating DV and did not cover education for the prevention of sexual violence [22]. In Japan, DV and sexual violence are not included in the educational guidelines for junior high school students or high school students, and implementation of preventive education is currently insufficient. However, adolescent physical, mental, and emotional development also involves an increasing interest in the opposite sex and sexuality, which is affected by various environments created by their surrounding human relationships and the Internet [19, 23]. Early implementation of violence prevention education using web-based learning for adolescents who are likely to be involved in a sexual relationship, before they become a perpetrator or victim of sexual violence, is essential to encourage the development of equal roles in male-female relationships. Cultivating attitudes that promote healthy conflict resolution is vital.

To lower the risk in 40 South African men and women who were at high risk for sexual violence and HIV transmission, a community-based intervention (SASA!) was implemented to improve communication skills between men and women [24]. Improvement in male-female relationships was assessed using an interview survey 2 months after the intervention program [24]. Another program—Creating Lasting Family Connections (CLFL) program—was implemented targeting 175 African American women to increase communication skills, relationship management, and rejection skills. This program reported an improvement in three skills and that violence from intimate partners had been reduced in the following 3 months when compared to the 44 persons in the control group [25]. Accordingly, social skills training utilized sexual violence prevention education for unmarried men and women in adolescence and adulthood to improve male-female relationships, and reducing violence is now considered effective both in Japan and abroad. However, there are still few empirical web-based learning studies that use online programs to prevent persons from becoming a perpetrator or a victim of sexual violence. Choi et al. implemented a program in 55 Korean Americans, the Korean Clergy for Healthy Families (KOCH) program, which included skills to enhance knowledge of intimate partner violence (IPV), methods to solve IPV, and self-efficacy in the context of IPV: 27 persons took this online program, and 28 were included as a control group. The intervention group was reported to have increased knowledge of IPV, self-efficacy, and also improved behavioral skills for prevention and intervention following the program; however, the difference between the two groups was not statistically significant [26]. Currently, health literacy is attracting attention in Japan as a means to maintain and improve the quality of life throughout the life course. Health literacy is the primary health power that collects, judges, executes, evaluates, modifies, and continues to execute health information; it is a skill to change lifestyles to be healthier, to reduce diseases and poor health conditions, and to live a comfortable life [27]. More recently, the need for sexual health literacy among adolescents who begin sexual behavior has been identified [28]. Through our web-based learning program, we would like to support adolescents in Japan and overseas to live a healthy and comfortable life by acquiring the needed knowledge, cultivating healthy attitudes for conflict resolution, and be able to implement them.

### Dangerous attitudes toward sexual violence that lead to mental and physical violence after taking DVD or web-based education program

Improvement in the dangerous attitudes that lead to sexual violence in adolescents was observed following the education program using DVD video teaching materials to prevent violence. This improvement effect was also observed for the web-based violence prevention learning program. According to the routine activity approach, crime stems from three factors: a motivated offender, a suitable target, and the absence of a capable guardian [9]. The risk of sexual violence in an adolescent’s daily routine, such as commuting to school, should be assumed. Studies in Japan of junior high school students report experiences of unwanted sexual harm in their daily routine, such as coerced sexual acts, groping, approached by a stranger, or stalking, with 12.7% of boys and 24.5% of girls reporting these experiences [12]. The number of recognized cases of indecent assault and actual status of sexual assault on men and women, as reported by the National Police Agency, is shown as data on the graph. In our educational program, the improvement effect is likely increased by using examples and videos that highlight the risk of sexual violence to both men and women in their daily routine, such as commuting and using the Internet.

### Preventive attitudes toward sexual violence that lead to mental and physical violence after taking DVD or web-based education program

Preventive attitudes toward sexual violence were strengthened in adolescents after the web-based learning program. A study suggested that women with low self-control experience more sexual violence [11]. Additionally, US studies have reported that sexual violence of female college students was significantly linked to low self-control and daily routine activities [11]. Previous strategies to improved attitudes in preventing community-based violence included education to enhance self-control in daily life, based on a slogan with impact and that would stick in children’s mind. Moreover, it was suggested that educational programs to prevent sexual violence web-based learning would strengthen preventive attitudes toward sexual violence.

### Research limitations and challenges

During the process to obtain study approval from the educational institution, the inclusion of a control group without any education program was not allowed. The Internet’s influence should also be considered, and it is conceivable that a web-based learning program might have been affected by the Internet learning environment and information from the Internet. Future studies should carefully consider interventional and assessment methods to minimize the potential influence of the Internet learning environment and information on the web. In terms of the research using DVD video teaching materials, there are limitations in the generalization of the results owing to the implementation of the questionnaire in a limited area. Further explanation of web-based learning programs and expansion to a wider range of subjects are needed. Consideration of the educational contents of the program and assessment methods that can be used both in Japan and overseas is required. In Japan and overseas, of the disruption that is being caused by the COVID-19, the development of a sexual violence prevention education program is needed.

## Conclusion

Implementation of an education program for adolescents using DVD video teaching materials and web-based learning to prevent sexual violence from an intimate partner, persons in the community, or through the Internet has been shown to improve the attitudes that lead to mental and physical violence and help them to cultivate attitudes that promote healthy conflict resolution. Improvement in the dangerous attitudes that lead to sexual violence was also observed. An education program using DVD video teaching materials or web-based learning may help prevent sexual violence among teens in Japan.

## Data Availability

The address of the home page is as follows: https://www.jrckicn.ac.jp/e-learning/
